# Magnetic anisotropy, unusual hysteresis and putative “up-up-down” magnetic structure in EuTAl_4_Si_2_ (T = Rh and Ir)

**DOI:** 10.1038/srep12021

**Published:** 2015-07-09

**Authors:** Arvind Maurya, A. Thamizhavel, S. K. Dhar, P. Bonville

**Affiliations:** 1 Department of Condensed Matter Physics and Materials Science, Tata Institute of Fundamental Research, Homi Bhabha Road, Colaba, Mumbai 400 005, India; 2 CEA, Centre d’Etudes de Saclay, DSM/IRAMIS/Service de Physique de l’Etat Condensé and CNRS UMR 3680, 91191 Gif-sur-Yvette, France

## Abstract

We present detailed investigations on single crystals of quaternary EuRhAl_4_Si_2_ and EuIrAl_4_Si_2_. The two compounds order antiferromagnetically at *T*_N1_ = 11.7 and 14.7 K, respectively, each undergoing two magnetic transitions. The magnetic properties in the ordered state present a large anisotropy despite Eu^2+^being an *S*-state ion for which the single-ion anisotropy is expected to be weak. Two features in the magnetization measured along the *c*-axis are prominent. At 1.8 K, a ferromagnetic-like jump occurs at very low field to a value one third of the saturation magnetization (1/3 M_0_) followed by a wide plateau up to 2 T for Rh and 4 T for Ir-compound. At this field value, a sharp hysteretic spin-flop transition occurs to a fully saturated state (M_0_). Surprisingly, the magnetization does not return to origin when the field is reduced to zero in the return cycle, as expected in an antiferromagnet. Instead, a remnant magnetization 1/3 M_0_ is observed and the magnetic loop around the origin shows hysteresis. This suggests that the zero field magnetic structure has a ferromagnetic component, and we present a model with up to third neighbor exchange and dipolar interaction which reproduces the magnetization curves and hints to an “up-up-down” magnetic structure in zero field.

The isothermal magnetization in an antiferromagnet at temperature *T* < *T*_N_, where *T*_N_ is the Néel temperature, increases with field from zero to full saturation at the spin-flip transition, when all the moments are aligned parallel to applied field. In the particular case where the field is parallel to the easy-axis along which the spins are pinned, the magnetization in an ideal collinear bipartite antiferromagnet is zero (*T* << *T*_N_) until the field induces a spin orientation, or spin-flop/metamagetic transition, leading to a sharp increase/saturation in magnetization. Often the spin-flop/metamagnetic transition is first order in nature as revealed by magnetic hysteresis when the polarity of the field is reversed. In all cases that we are aware of, the magnetization of an antiferromagnet decreases to zero when the field is reduced to zero. In contrast, the magnetization of a ferromagnet shows a hysteretic behaviour around the origin, characterized by a finite remanence and a coercive field. Among other factors, the single-ion magnetocrystalline anisotropy plays an important role in the coercive field of a ferromagnet. Here, we report, to our knowledge the very first case, the observation of hysteresis around the origin in two antiferromagnets, which are thought to present a peculiar magnetic structure. The unusual nature of our observation is further heightened by the fact that the two antiferromagnets EuTAl_4_Si_2_ (T = Rh and Ir) derive their magnetism from Eu^2+^which is an *S*-state ion (*S* = 7/2; *L* = 0). For *S*-state rare earth ions like Eu^2+^and Gd^3+^, the single-ion magnetocrystalline anisotropy is usually quite small.

The synthesis of single crystals of new quaternary compounds EuTAl_4_Si_2_ (T = Rh and Ir), using the Al-Si binary eutectic as flux, has recently been reported^1^. The two compounds were found to adopt an ordered derivative of the ternary KCu_4_S_3_-type tetragonal, *tP*8, *P*4/*mmm* structure. The Eu, Al, Si and T atoms occupy respectively the K(1*a*), Cu(4*i*), S2(2*h*) and S1(1*a*) Wyckoff sites, which leads to quaternary and truly stoichiometric 1:1:4:2 compounds. The local symmetry at the Eu site is fourfold axial (4/*mmm*). Preliminary magnetization data^1^, taken with the applied field parallel to [001] direction, revealed a divalent state for Eu ions in both compounds and a magnetic transition at ≈12 and ≈15 K in the Rh and Ir analogs, respectively. Electronic structure calculations using the local spin density approximation^1^ are in conformity with the divalent state of Eu ions.

In the present work, we probe in detail the magnetic behavior of these two intermetallic compounds along the principal crystallographic directions of the tetragonal cell, using the techniques of magnetization, heat capacity and electrical transport. Additional information about the magnetic transitions is derived from ^151^Eu Mössbauer spectroscopy. We find a rather unusual and highly anisotropic magnetic response, which points to a probable ferrimagnetic-like spin arrangement, scarcely observed in intermetallics with divalent Eu. We show that such a ground magnetic structure can be predicted using a mean field model with three exchange integrals.

## Results

### Magnetic susceptibility

**Figure 1:**
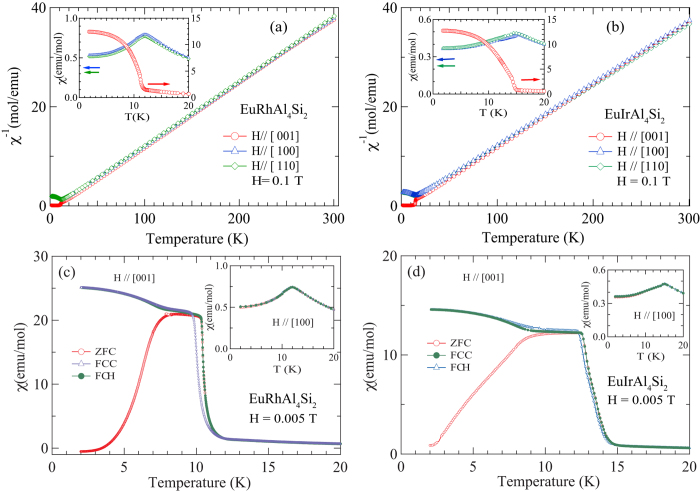
Inverse magnetic susceptibility of (**a**) EuRhAl_4_Si_2_ and (**b**) EuIrAl_4_Si_2_ along [001], [100] and [110] crystallographic directions. The insets show the anisotropy in the low temperature susceptibility. (**c**–**d**) The magnetic susceptibility in a field of 0.005 T: zero field cooled (ZFC), field cooled cooling (FCC) and field cooled heating (FCH) data for *H* ‖ [100] and *H* ‖ [001].

Figures. 1(a,b) show the inverse susceptibility data between 300 and 1.8 K respectively for EuRhAl_4_Si_2_ and EuIrAl_4_Si_2_ in a field of 0.1 T applied along the [001], [100] and [110] directions. The molar susceptibility follows the Curie-Weiss law:


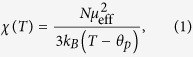


where *N* is the Avogadro number, almost in the entire paramagnetic region. The effective moment *μ*_e*ff*_ and the Curie-Weiss temperature *θ*_*p*_ are listed in Table 1. The *μ*_e*ff*_ values in both compounds are comparable to the Hund’s rule derived value of 7.94 *μ*_B_ for free-ion Eu^2+^. The positive *θ*_p_ values in these *a priori* antiferromagnetic (AF) materials indicate the presence of competing nearest neighbor and next-nearest exchange couplings, with opposite signs. This is probably brought about in these two metallic compounds by the oscillatory RKKY (Ruderman-Kittel-Kasuya-Yosida) exchange interaction. An appreciable anisotropy in the susceptibility, plotted between 1.8 and 20 K in the insets of Fig. 1, develops at low temperatures in the two compounds. It is clearly evidenced by the *χ*(*T*) data below 20 K, taken in a low field of 0.005 T, shown in Figs. 1(c,d) for *H* ‖ [100] and *H* ‖ [001].

The susceptibility *χ*_*a*_(*T*) of EuRhAl_4_Si_2_ along the [100] direction (inset, Fig. 1(c)) shows a peak at 12 K followed by a minor kink at 10.4 K (which correspond closely with the peak in zero field heat capacity at 11.7 K and the small kink at 10.4 K, *vide infra*) with nearly overlapping ZFC and FC data, characteristic of an AF transition. In EuIrAl_4_Si_2_, (inset, Fig. 1(d)), the corresponding data show a peak at nearly 15 K, followed by a barely discernible anomaly in the 13–14 K range (which also corresponds closely with the sharp peak in zero field heat capacity at 14.7 K, followed by a peak at 13.2 K, *vide infra*). Data taken with *H* ‖ [110] (not shown) reflect a nearly isotropic magnetic response in the *ab*-plane in both compounds.

On the other hand, along the tetragonal *c*-axis [001], the susceptibility *χ*_*a*_(*T*) exhibits ferromagnetic-like behavior, with its peak value more than an order of magnitude larger than the peak value of *χ*_*a*_(*T*) (main panel Figs. 1(c,d)). There is a substantial thermomagnetic irreversibility between ZFC and FC runs, which decreases as the applied field is increased (data at higher fields not shown here for the sake of clarity). Phenomenologically, the ZFC/FC behavior is similar to that seen in ferromagnets with a sizeable coercivity. The upturn in *χ*_*c*_(*T*) begins at nearly the same temperature as the peak in *χ*_*a*_(*T*). Data were taken in both field-cooled-cooling (FCC) and field-cooled-heating (FCH) modes. The observation of hysteresis in these two plots is in line with the ferromagnetic character of the transition for *H* ‖ [001].

**Figure 2:**
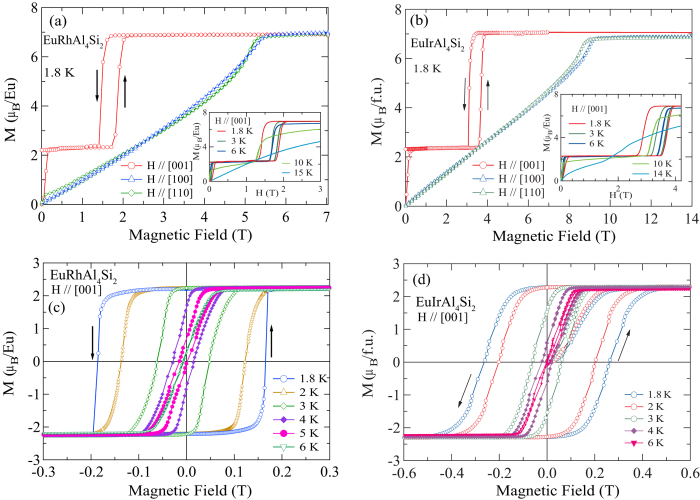
*M* vs *H* for (**a**) EuRhAl_4_Si_2_ and (**b**) EuIrAl_4_Si_2_ at 1.8 K for *H* in the *ab*-plane and along [001] and at selected temperatures for *H* along [001](insets) (**c**). Low field hysteresis showing the coercive field in EuRhAl_4_Si_2_ (**c**) and EuIrAl_4_Si_2_ (**d**).

The behavior of the in-field magnetization at 1.8 K, measured in the *ab*-plane along [100] and [110], and along [001] (Figs. 2(a,b)), is in conformity with the *χ*(*T*) data depicted in Figs. 1(c,d). For *H* in the *ab*-plane, the magnetisation in EuRhAl_4_Si_2_ increases almost linearly, reaching a (spin-flip) saturation field near 5.5 T, with a saturated Eu^2+^ moment M_0_ = 7 *μ*_B_, as expected for a *g* = 2, *S* = 7/2 ion. This behavior is characteristic for an AF structure where the moments are perpendicular to the field. At higher temperatures, both the spin-flip field value and the saturation magnetization decrease (data not shown). By contrast, for *H* ‖ [001], there is a jump of the magnetization at very low field to a value 2.3 *μ*_B_, which is almost exactly 1/3 of M_0_. The magnetization plateau is broken near 1.7–2 T by a hysteretic spin-flop jump to saturation. This behavior tells that the Eu moment probably lies along the *c*-axis, and the low field jump hints to the presence of an average ferromagnetic component along *c* in the zero field structure. The 1/3 M_0_ value suggests that the latter could consist in a motif of 3 spins along *c*, two pointing up and one pointing down. The zero field magnetic structure would then consist in ferromagnetic planes with moments along *c* in an “up-up-down” sequence. The inset of Fig. 2(a) shows that the width of the hysteresis associated with the spin-flop jump decreases and it occurs at lower fields with the increase of temperature. The Ir sibling (Fig. 2(b)) shows exactly the same behavior, but with a spin-flop field of 3.9–4 T along [001] and a spin-flip field of 9.2 T perpendicular to [001]. Figures 2(c,d) show the hysteretic behaviour of the magnetisation in EuRhAl_4_Si_2_ and EuIrAl_4_Si_2_ respectively for magnetisation jump at *H* close to origin. Surprisingly, there is a sizeable remanent magnetization of 2.3 *μ*_B_/f.u. at 1.8 K and a large coercive field of nearly 0.18 T and 0.25 T in the Rh and Ir analogue, respectively. The width of the loop depends sensitively on the temperature and it has decreased by more than half at 3 K, compared to its value at 1.8 K and by 6 K the hysteresis has practically vanished. We suggest hysteresis is due to the presence of a ferromagnetic component in the zero field structure of these compounds, the possible domains having their mean magnetization pointing either up or down. The coercive field would then correspond to a complete reversal of the domains magnetization along the direction of the field. The primary sources of coercivity are the magnetocrystalline anisotropy and the dipolar field, which has been shown to play an important role in Eu compounds with low transition temperatures^2^. The rather high coercive field of 0.2 T at 1.8 K points thus to an unusually high crystalline anisotropy for an *L* = 0 divalent Eu ion, for which it vanishes in principle at first order. This is supported by the modelling of the magnetic behavior presented in the discussion section. Magnetic characteristics of EuRhAl_4_Si_2_ and EuIrAl_4_Si_2_ are summarized in Table 2.

### Heat Capacity

**Figure 3:**
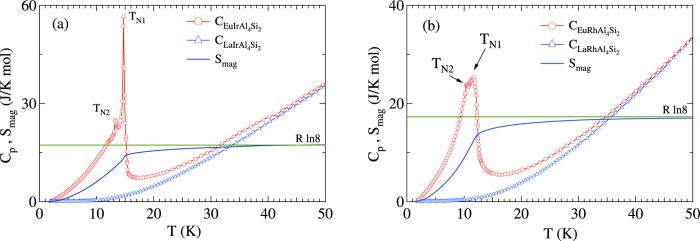
Heat capacity *C*_*p*_ and entropy *S*_*mag*_ as a function of temperature in EuTAl_4_Si_2_ and nonmagnetic LaTAl_4_Si_2_ for (**a**) T = Ir and (**b**) Rh. The horizontal line depicts the high temperature limit (*R* In 8) of the magnetic entropy for an *S* = 7/2 spin system.

The heat capacity of the two compounds is shown in Fig. 3. We have also plotted the heat capacity of the corresponding La analogs, which serves as a measure of the phonon contribution assuming that the phonon spectra in the La and corresponding Eu compound are identical. In EuIrAl_4_Si_2_, an extremely sharp peak with a peak value of nearly 60 J/mol K and peak position of 14.7 K (Fig. 3(a)), indicative of a first order phase transition from the paramagnetic state to an intermediate magnetically ordered state, is followed by a second transition near 13.2 K marked by a small anomaly at that temperature. The heat capacity of EuRhAl_4_Si_2_ (Fig. 3(b)) is apparently marked by only one peak at 11.7 K which is relatively less sharp and has a lower peak height. However, a small kink at 10.4 K is discernible; both are marked by arrows in the figure. The ^151^Eu Mössbauer spectra, to be described below, also show the presence of two magnetic transitions in both compounds. The magnetic contribution to the heat capacity, *C*_*mag*_, was obtained by subtracting the normalized heat capacity of the corresponding La compound, following the procedure described in Ref. 3. The entropy *S*_*mag*_ calculated by integrating *C*_*mag*_/*T* against the temperature is also shown in Figs. 3(a,b). Nearly 75% of the full entropy *R* In 8 is recovered at the transition temperature, and the remaining by 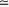
40 K.

### Electrical Resistivity and Magnetoresistance

**Figure 4:**
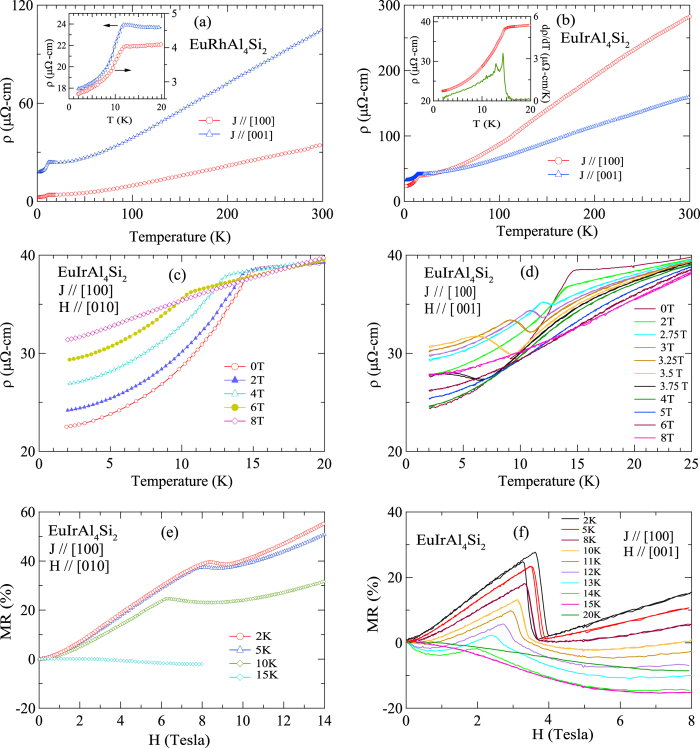
Electrical resistivity as a function of temperature when the current is parallel to major crystallographic directions in (**a**) EuRhAl_4_Si_2_ and (**b**) EuIrAl_4_Si_2_. *ρ*(*T*) data as a function of temperature at selected magnetic fields applied along (**c**) [010] and (**d**) [001], and field evolution of *MR* for (**e**) *H* ‖ [010] and (**f**) *H* ‖ [001] in EuIrAl_4_Si_2_ with current being parallel to [100].

Figures 4(a,b) depict the variation of electrical resistivity *ρ*(*T*) with temperature, which reveals a normal metallic nature for the two compounds. The data have been taken with the current density along [100] and [001], respectively. The resistivity is anisotropic which indicates an anisotropic Fermi surface of these two compounds with tetragonal symmetry. The fall in the resistivity at *T*_N1_, due to the gradual loss of spin disorder scattering below the magnetic transition is clearly visible, while *T*_N2_ is marked by only a barely discernible kink in the *ρ*(*T*) plot but clearly observed in the derivative plot as shown for EuIrAl_4_Si_2_. To within the precision of our measurements we did not detect any hysteretic behavior of the resistivity when the temperature was cycled up and down across the magnetic transition.

The variation of resistivity with temperature in the magnetically ordered state in applied magnetic fields, and the magnetoresistance *MR* derived from the data can be qualitatively understood on the basis of the magnetization data discussed above. Figures 4(c,d) show the resistivity below 25 K in EuIrAl_4_Si_2_ with *J* ‖ [100] and *H* ‖ [010] and [001] at selected values of field between 0 and 8 T. In Fig. 4(c) the main anomaly in the resistivity occurring at *T*_N1_ shifts towards lower temperatures with increasing field as expected for an antiferromagnet. The anomaly almost disappears at 8 T as the spin-flip field value is approached.

The Rh analog also shows similar behavior except that the anomaly at *T*_N1_ vanishes at a lower field consistent with a lower spin-flip field in this compound when the field is applied in the *ab*-plane. In Fig. 4(d), which shows the data for *H* ‖ [001], the main anomaly at *T*_N1_ again shifts to lower temperature with field. Additionally, the resistivity at 2.75, 3, 3.25, 3.5 and 3.75 T shows an upturn at a temperature that is dependent on the applied field. An upturn, prima-facie suggests a gap-opening; neutron scattering is required to probe if there is a change in the magnetic symmetry with field. The magnetoresistance defined as *MR* = Δ*R*/*R*(0), where Δ*R* = *R*(*H*) − *R*(0), is shown in Figs. 4(e,f) for EuIrAl_4_Si_2_. The *MR* of the Rh-analogue is qualitatively similar (data shown in Supplementary information). The *MR* of an antiferromagnetic material below *T*_N_ is expected to be positive as the applied field tends to break the antiferromagnetic couplings thereby increasing the spin disorder scattering. Indeed, the *MR* is positive below *T*_N_ in both the compounds and increases with increasing field. Near the field-induced spin-flip transition, the *MR* tends to be field independent (Fig. 4(e)). However, at higher fields the positive *MR* keeps on increasing though at a somewhat slower rate. In this context it may be mentioned that the *MR* of non-magnetic reference compound LaIrAl_4_Si_2_ is positive and nearly 20% at 14 T; indicating a positive *MR* due to background conduction electrons. With the increase of temperature the spin-flip field decreases and this is reflected well in the *MR* data of EuIrAl_4_Si_2_ at 2, 5 and 10 K, respectively. Just above *T*_N1_ at 15 K the *MR* of EuIrAl_4_Si_2_ in the paramagnetic state is relatively small and negative in almost the entire range up to 8 T. The negative, albeit small, *MR* at 15 K may be due to the combined partial quenching of the spin fluctuations above *T*_N1_ and positive MR due to background conduction electrons.

The *MR* for *H* ‖ [001] and *J* ‖ [100] at selected values of temperature in fields up to 8 T is shown in Fig. 4(f). The prominent feature is the sharp drop and hysteresis in *MR* at fields where the metamagnetic jump is seen in the magnetization of the Ir compound. The hysteresis is reduced in width and occurs at lower fields with the increase in temperature, in excellent correspondence with the magnetization data for *H* ‖ [001] (see, Fig. 2(b) inset). Thus there is a close correlation between the magnetization and the *MR* data which clearly reveal the first order nature of the metamagnetic transition. Qualitatively the behavior of *MR* in the two compounds is similar. At low fields, below *T*_N_, it is positive and increases with field. Above the metamagnetic transition the *MR* increases with field, its magnitude and sign depending upon the temperature and field. In the paramagnetic state at 15 K in EuRhAl_4_Si_2_ and at 20 K in EuIrAl_4_Si_2_, the *MR* is negative at all fields.

### ^151^Eu Mössbauer spectra

**Figure 5:**
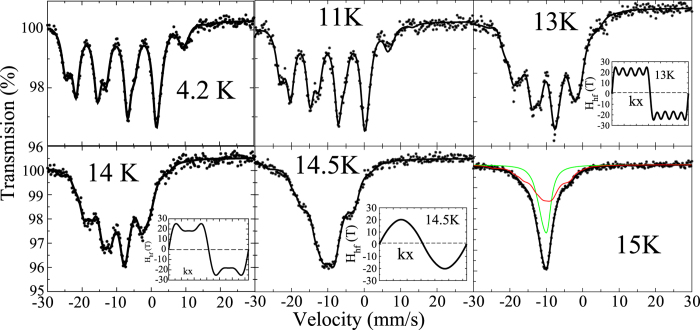
^151^Eu Mössbauer spectra in EuIrAl_4_Si_2_ between 4.2 K and 15 K (see text). The modulations at 13 K, 14 K and 14.5 K along the propagation vector **k** are shown next to the corresponding spectrum.

Additional information on the two compounds was obtained by employing ^151^Eu Mössbauer spectroscopy. Spectra were recorded between 4.2 and 15 K. We show here only the data for T = Ir (the spectra for the Rh-analogue are shown in the Supplementary information). We recall that the specific heat data show a transition near 14.7 K followed by another one at 13.2 K. At 4.2 K, the spectrum is a standard Eu^2+^ hyperfine pattern with a single hyperfine field of 30.1(1) T, meaning that the magnetic structure is equal moment. The spectra start changing shape near 13 K, and Fig. 5 shows their rapid evolution in the small temperature interval 13–15 K. At 13, 14 and 14.5 K, the spectra are purely incommensurate modulation (ICM) patterns, and at 15 K the spectrum is a superposition of an ICM spectrum (red line) and of a single line paramagnetic spectrum (green line). Above 15 K, the spectrum is a single line characteristic of the paramagnetic phase. Although the transitions we observe by Mössbauer spectroscopy are slightly shifted with respect to those given by the specific heat peaks, the spectra illustrate well the way EuIrAl_4_Si_2_ goes, on cooling, from the paramagnetic phase to the ICM phase, then to the commensurate lock-in phase, the first transition being first order as witnessed by the coexistence of the two phases in the spectra at 15 K. The spectra in EuRhAl_4_Si_2_ are very similar, the transitions being shifted to somewhat lower temperature, in agreement with the specific heat data indicating a first transition close to 11.7 K, followed by another at 10.4 K. This cascade of transitions is a rather common feature in intermetallics with divalent Eu or trivalent Gd ions^3^. In particular, it is present in EuPdSb^4^ and has been discovered more recently in EuPtSi_3_^5^ and EuNiGe_3_^2^. It seems to be concomitant with the occurence of a sizeable anisotropy of the magnetisation, as in the present materials.

## Discussion

Two noticeable features in the magnetization along the *c*-axis in both compounds are the existence of a jump at low field followed by a plateau region with magnetization 1/3 M_0_ (M_0_ = 7 *μ*_B_/f.u. at 2 K). In some rare earth antiferromagnets, plateaus in the magnetization along the easy-axis have been reported, but at a finite field value due to the strong crystal field induced Ising character of the considered rare earth^6^. Some examples are DyCo_2_Si_2_ (*T*_N_ = 24 K)^7^,^8^, orthorhombic DyCu_2_ (*T*_N_ = 31 K)^9^ and CeZn_2_ (*T*_N_ = 7 K)^10^. The intermediate phases at the plateaus correspond to successive flipping of blocks of magnetic moments which change the magnetic periodicity and the propagation vector. In hexagonal PrRh_3_B_2_, which shows the intermediate 1/3M_0_ state, it was proposed that the ferromagnetic linear chains along [0001] are coupled antiferromagnetically, which leads to an Ising-spin structure in a triangular lattice^11^.

To our knowledge, the only very low field 1/3 M_0_ plateau has been observed at very low temperature for a field along [111] in spin-ice Dy_2_Ti_2_O_7_^12^, which does not undergo magnetic ordering. In this case, the plateau is due to the peculiar pyrochlore lattice structure with extreme Ising anisotropy at each of the 4 Dy sites having threefold symmetry along one of the 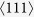 axes.

In EuRhAl_4_Si_2_ and EuIrAl_4_Si_2_, the presence of such an unusual low field plateau is probably due to a combination of a low (but sizeable) crystalline anisotropy and of a peculiar ferrimagnetic-like magnetic structure, which we believe consists in a tripled magnetic unit cell along *c* with “up-up-down” moment arrangement. The propagation vetor would then be *k* = (0 0 1/3). Neutron diffraction experiments on a single crystal of EuRhAl_4_Si_2_ are planned in order to check the existence of such a structure.

We present in the following a model which sets our assumption of an “up-up-down” sequence, with moments along *c*, on a firmer ground. The model considers a magnetic super-cell with 6 sub-lattices consisting each of a ferromagnetic plane perpendicular to [001]. The planes are bi-dimensional simple cubic lattices with parameter *a*, and the distance between planes is *c*. The Eu ions are taken to interact *via* the short range RKKY exchange and the infinite range dipolar interaction, and they are submitted to a crystalline anisotropy of the form: 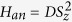, where *Oz* is the c-axis, appropriate for axial symmetry around c. As underlined in Refs 2,13, inclusion of the dipolar interaction is essential to reproduce the magnetic behaviour in a realistic way in compounds with *S*-state Eu^2+^ and Gd^3+^ ions because it competes with the crystalline anisotropy for establishing the moment direction and the spin-flop fields. We detail in the following these three interactions:

i) we consider that each Eu ion interacts with its neighbours through 3 exchange integrals: in-plane *J*_0_ (4 nearest neighbour ions at distance *a*), and interplane (along *c*) *J*_1_ (2 first neighbour ions at distance *c*) and *J*_2_ (2 second neighbour ions at distance 2*c*). We treat the exchange in the molecular field approximation, i.e. the exchange field 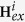 on a given spin **S**_*i*_ writes:


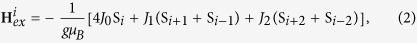


where the indexes must be taken mod. 6. Due to the oscillatory character of the RKKY interaction, the integrals can be positive (ferromagnetic) or negative (antiferromagnetic).

ii) the infinite range dipolar field on each ion contains two contributions: the field originating from the ion own sub-lattice and that coming from each of the 5 other sub-lattices. The field acting on an ion in sublattice *i* can be written: 
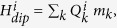
 where 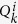 is a matrix containing lattice sums evaluated using Ewald-type summations^14^, *k* runs over all the sublattices and m_*k*_ is the moment in the *k*th sublattice (in units of *μ*_*B*_). In units of the Lorentz field 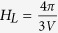, with *V* = 6*a*^2^*c* being the volume of the chosen magnetic cell, the own ion dipolar matrix writes:


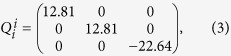


and the 5 other matrices are equal with only one non-zero matrix element: 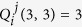 in the same units. For EuRhAl_4_Si_2_, *a* = 0.418 nm and *c* = 0.828 nm so that *H*_*L*_ = 44.76 G. Since *m*_0_ = 7 *μ*_*B*_, the dipolar fields are of the order of 0.3 T.

iii) the crystalline anisotropy term 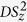 would create, in the absence of dipolar field, an easy axis along [001] (c-axis) for *D* < 0. In the presence of the dipolar field, we find that *D* must be taken rather large (negative) in order to have moments along *c*, otherwise the dipolar field forces the moment to lie in the (*ab*) plane.

**Figure 6:**
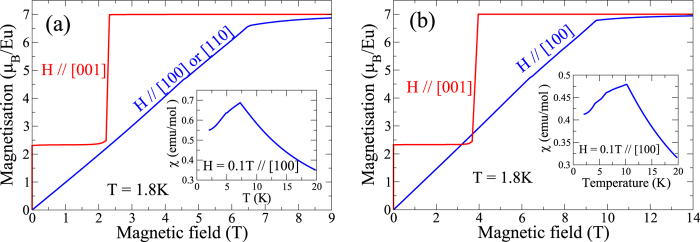
Calculated isothermal magnetization curves at 1.8 K for *H* || [001] and in the (*ab*) plane, and, in the insets, calculated susceptibility for *H* || [100]: **(a)** with *J*_0_ = 0.18 K, *J*_1_ = −0.02 K, *J*_2_ = −0.17 K and *D* = −1.3 K, to be compared with data in EuRhAl_4_Si_2_ of Figs. 1(a) and 2(a) and (**b**) with *J*_0_ = 0.22 K, *J*_1_ = −0.025 K, *J*_2_ = −0.29 K and *D* = −1.5 K, to be compared with data in EuIrAl_4_Si_2_ of Figs. 1(b) and 2(b).

In order to obtain the magnetisation and the susceptibility, we perform for each sub-lattice an exact diagonalisation of the hamiltonian:


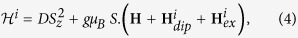


where **H** is the applied magnetic field, and obtain 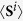 which is treated self-consistently within the 6 sublattices until convergence is reached. A scan in the 4 parameter space {*J*_0_, *J*_1_, *J*_2_, *D*} was performed, taking the nearest neighbour in-plane exchange integral *J*_0_ positive (ferromagnetic), ensuring ferromagnetic planes normal to [001], and *D* large negative, ensuring the c-axis as easy axis. We could find for both compounds a point around which the zero field ground state is the “up-up-down” structure, and for which the magnetisation curves are in rather good agreement with experiment as to the low field jump, the spin-flop and spin-flip fields. However, the *T*_N_ values are somewhat lower (by 2–3 K) than the actual values. For EuRhAl_4_Si_2_, these parameters are: *J*_0_ = 0.18 K, *J*_1_ = −0.02 K, *J*_2_ = −0.17 K and *D* = −1.3 K. The corresponding *m*(*H*) and *χ*(*T*) curves are shown in Fig. 6(a), where it can be seen that 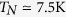, instead of the experimental value 10–11 K. Similarly, the parameter set: *J*_0_ = 0.22 K, *J*_1_ = −0.025 K, *J*_2_ = −0.29 K and *D* = −1.5 K reproduces well the data in EuIrAl_4_Si_2_ (see Fig. 6(b)), but with *T*_N_ = 10 K instead of 13–14 K. For this latter compound, *J*_0_ and 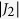 are larger than for EuRhAl_4_Si_2_, which accounts for the higher Néel temperature and characteristic field values. It may be noted that *J*_1_ is close to zero, which could correspond to a node of the RKKY oscillating function, and that the crystalline anisotropy coefficient *D* is unusually large in these two materials. Indeed, although this quantity is seldom measured, its expected order of magnitude for Eu^2+^ or Gd^3+^ is 0.1 K^15^. We presently have no explanation for this enhanced value.

## Conclusion

We have performed a series of measurements to probe in detail the magnetic properties of single crystals of two tetragonal quaternaries, EuRhAl_4_Si_2_ and EuIrAl_4_Si_2_. Both present a first order transition from a paramagnetic to an incommensurate antiferromagnetic phase at 11.7 and 14.7 K respectively, followed by a lock-in transition to a commensurate antiferromagnetic phase at 10.4 and 13.2 K. Such a transition cascade is rather common among Eu intermetallics. By contrast, the magnetic behavior deep in the antiferromagnetic phase is rather unexpected for isotropic divalent Eu: the response is antiferromagnetic for a field applied in the *ab*-plane and ferromagnetic for a field applied along *c*. In this latter case, the magnetization shows a low field jump followed by an intermediate plateau region attaining a value of 1/3 M_0_. Beyond the plateau region there is a sharp hysteretic spin-flip transition. Our in-field transport data correlate well with this behaviour of the magnetisation. Surprisingly, the magnetization shows a substantial hysteresis around the origin which is not expected in divalent Eu compounds. The magnetization data hint to the presence of an unusual “up-up-down” zero field magnetic structure which is shown to be obtainable by the mean field model presented here, but which must be confirmed by neutron diffraction experiments.

## Methods

The details of the single crystal growth and structure have been reported in Ref. 1. In the present work single crystals of LaTAl_4_Si_2_ (T = Rh and Ir), isostructural to Eu analogs, were also grown following the same protocol as adopted for the Eu compounds. The flux-grown crystals were oriented by means of the Laue diffraction technique in back-reflection mode and cut appropriately using a spark erosion cutting machine. The magnetization as a function of temperature and magnetic field was measured in Quantum Design SQUID and VSM magnetometers between 1.8 and 300 K. Heat capacity and electrical resistivity in zero and applied fields were measured in a Quantum Design PPMS. ^151^Eu Mössbauer absorption spectra were taken at selected temperatures using a commercial ^151^SmF_3_ *γ*-ray source, mounted on a constant acceleration electromagnetic drive, in a standard liquid He cryostat.

## Additional Information

**How to cite this article**: Maurya, A. *et al.* Magnetic anisotropy, unusual hysteresis and putative "up-up-down" magnetic structure in EuTAl_4_Si_2_ (T = Rh and Ir). *Sci. Rep.* **5**, 12021; doi: 10.1038/srep12021 (2015).

## Supplementary Material

Supplementary Information

## Figures and Tables

**Table 1:** Effective moment and paramagnetic Curie temperature values in EuRhAl_4_Si_2_ and EuIrAl_4_Si_2_ along the major crystallographic directions.

	EuRhAl_4_Si_2_		EuIrAl_4_Si_2_	
	*μ*_*eff*_ (*μ*_B_/*f*.*u*.)	*θ*_*p*_(*K*)	*μ*_*eff*_ (*μ*_B_/*f*.*u*.)	*θ*_*p*_(*K*)
*H* ‖ [100]	7.64	8.0	7.78	3.0
*H* ‖ [110]	7.84	6.9	8.06	2.3
*H* ‖ [001]	7.83	10.4	7.90	9.2

**Table 2:** Magnetic characteristics of EuRhAl_4_Si_2_ and EuIrAl_4 _Si_2_.

	*T*_N1_	*T*_N2_	Spin flip field at 1.8 K (T)		Hysteresis at 1.8 K *H* ‖*c*	
			*H* ‖*ab*-plane	*H*‖*c*	*H*_*c*_ (*T*)	Plateauwidth (T)
EuRhAl_4_Si_2_	11.7	10.4	5.5	1.8	0.18	1.2
EuIrAl_4_Si_2_	14.7	13.2	9.2	3.9	0.25	2.5
