# Supplementary material for: Magnetic anisotropy, unusual hysteresis and putative “up-up-down” magnetic structure in EuTAl4Si2 (T = Rh and Ir)
Source: Sci Rep. 2015 Jul 9;5:12021. doi: 10.1038/srep12021 (PMC4496665; doi:10.1038/srep12021)
Supplement: Supplementary Information [file srep12021-s1.pdf]

Supplementary Information

Magnetic anisotropy, unusual hysteresis and putative "up-up-down" magnetic structure in $EuTAl_4Si_2$ (T = Rh and Ir)

Arvind Maurya,[1] A. Thamizhavel,[1] S. K. Dhar,[1,*] and P. Bonville[2]

[1]*Department of Condensed Matter Physics and Materials Science, Tata Institute of Fundamental Research, Homi Bhabha Road, Colaba, Mumbai 400 005, India*

[2]*CEA, Centre d'Etudes de Saclay, DSM/IRAMIS/Service de Physique de l'Etat Condensé and CNRS UMR 3680, 91191 Gif-sur-Yvette, France*

(Dated: May 7, 2015)

* sudesh@tifr.res.in

Electrical resistivity of $EuRhAl_4Si_2$ in selected magnetic fields

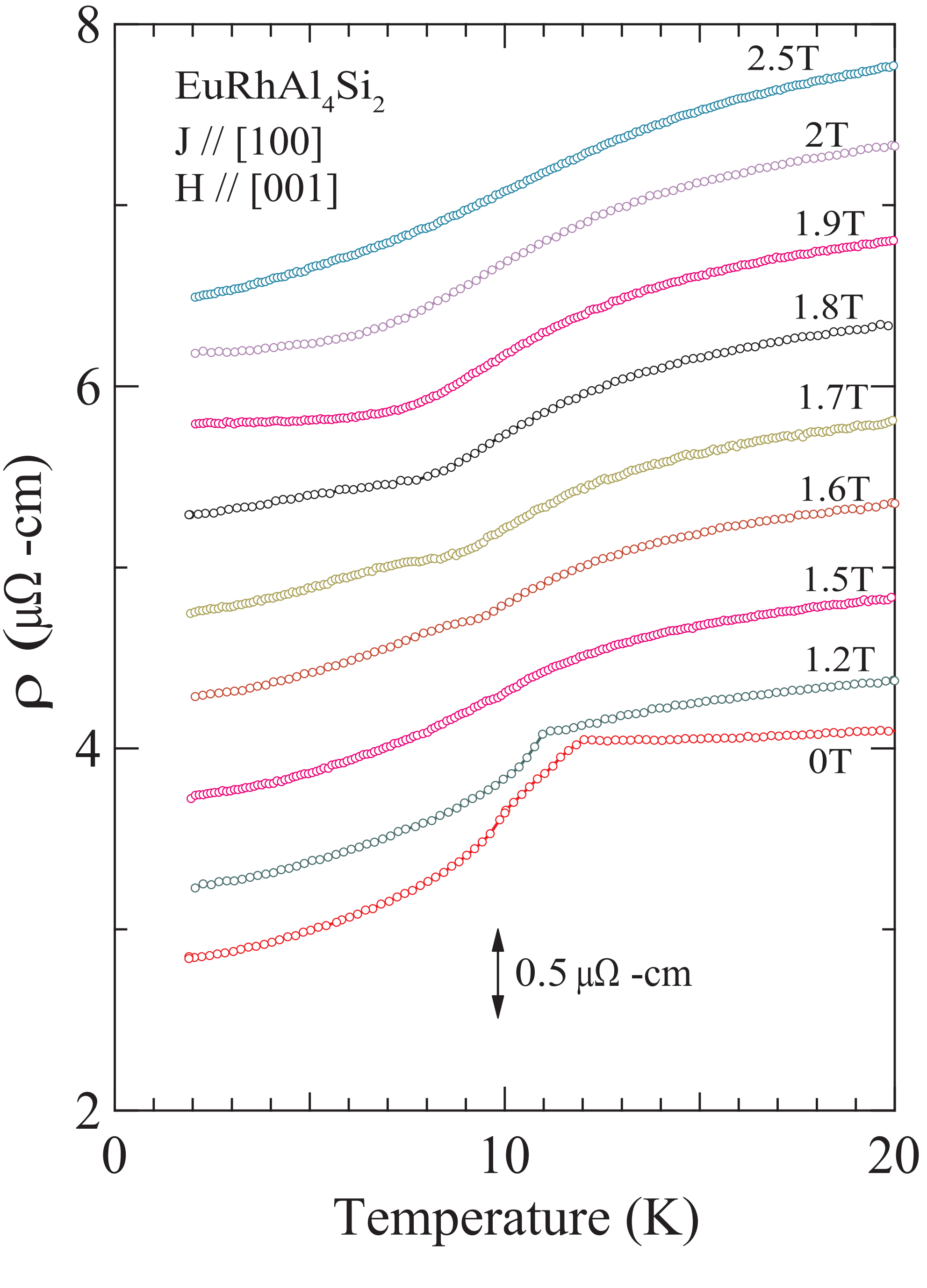

FIG. 1. Resistivity of $EuRhAl_4Si_2$ below 20 K at selected fields for $J \parallel [100]$ and $H \parallel [001]$. Traces other than for zero field have been shifted upward for clarity.

^{151}Eu Mössbauer spectra in $EuRhAl_4Si_2$

We recall that the specific heat data show a transition near 11.7 K probably followed by another one at 10.4 K. At 4.2 K, the spectrum is a standard Eu^{2+} hyperfine pattern with a single hyperfine field of 30.2(1) T, meaning that the magnetic structure is equal moment commensurate. The spectra start changing shape at 11 K, and Figure 2 shows their rapid evolution in the small temperature interval 11-12.5 K. At 11 K, a superposition of a single hyperfine field spectrum (82%, blue line) and of an incommensurate modulation (ICM) spectrum (18%, red line) is observed. At 11.5 and 12 K, the spectra are purely ICM patterns, and at 12.5 K the spectrum is a superposition of an ICM spectrum (72%, red line) and of a single line paramagnetic spectrum (28%, green line). At 14 K, the spectrum is a single line characteristic of the paramagnetic phase. Although the transitions we observe by

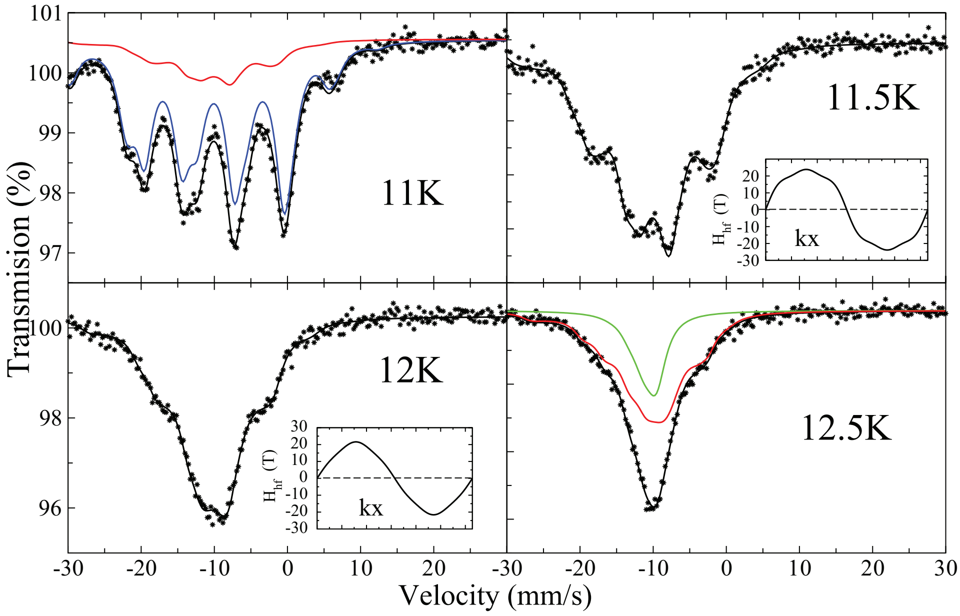

FIG. 2. ^{151}Eu Mössbauer spectra in $EuRhAl_4Si_2$ between 11 K and 12.5 K (see text). The modulations at 11.5 K and 12 K along the propagation vector **k** are shown next to the corresponding spectrum.

Mössbauer spectroscopy are slightly shifted (by 0.5 K) with respect to those given by the specific heat peaks, the spectra illustrate well the way $EuRhAl_4Si_2$ goes, on cooling, from the paramagnetic phase to the ICM phase, then to the commensurate lock-in phase, both transitions being first order as witnessed by the coexistence of the two phases in the spectra.
